# Loss of membrane integrity drives myofiber death in lipin1‐deficient skeletal muscle

**DOI:** 10.14814/phy2.14620

**Published:** 2020-10-28

**Authors:** Sandhya Ramani Sattiraju, Abdulrahman Jama, Abdullah A. Alshudukhi, Nicholas Edward Townsend, Daniel Reynold Miranda, Rebecca R Reese, Andrew A. Voss, Hongmei Ren

**Affiliations:** ^1^ Department of Biochemistry and Molecular Biology Wright State University Dayton OH USA; ^2^ Department of Biological Sciences Wright State University Dayton OH USA

**Keywords:** apoptosis, lipin1, membrane integrity, necrosis, skeletal muscle

## Abstract

Mutations in lipin1 are suggested to be a common cause of massive rhabdomyolysis episodes in children; however, the molecular mechanisms involved in the regulation of myofiber death caused by the absence of lipin1 are not fully understood. Loss of membrane integrity is considered as an effective inducer of cell death in muscular dystrophy. In this study, we utilized a mouse line with selective homozygous lipin1 deficiency in the skeletal muscle (Lipin1^Myf5cKO^) to determine the role of compromised membrane integrity in the myofiber death in lipin1‐deficient muscles. We found that Lipin1^Myf5cKO^ muscles had significantly elevated proapoptotic factors (Bax, Bak, and cleaved caspase‐9) and necroptotic proteins such as RIPK1, RIPK3, and MLKL compared with WT mice. Moreover, Lipin1^Myf5cKO^ muscle had significantly higher membrane disruptions, as evidenced by increased IgG staining and elevated uptake of Evans Blue Dye (EBD) and increased serum creatine kinase activity in Lipin1^Myf5cKO^ muscle fibers. EBD‐positive fibers were strongly colocalized with apoptotic or necroptotic myofibers, suggesting an association between compromised plasma membrane integrity and cell death pathways. We further show that the absence of lipin1 leads to a significant decrease in the absolute and specific muscle force (normalized to muscle mass). Our work indicates that apoptosis and necroptosis are associated with a loss of membrane integrity in Lipin1^Myf5cKO^ muscle and that myofiber death and dysfunction may cause a decrease in contractile force.

## Graphical abstract

Our data show that loss of membrane integrity is strongly associated with necroptosis and apoptosis in the lipin1 deficient muscle.

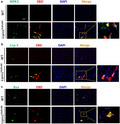

## 1 INTRODUCTION

Lipin1 is a phosphatidic acid (PA) phosphatase (PAP) which catalyzes the conversion of PA to diacylglycerol (DAG), a critical step in the synthesis of glycerophospholipids (Csaki and Reue, (2010); Reue & Zhang, 2008). It accounts for most of the PAP activity in skeletal muscle (Donkor et al., 2007). Clinical studies have suggested that patients with compound heterozygous lipin1 mutations exhibit muscle wasting in glycolytic muscle fibers and massive rhabdomyolysis episodes in early life (Michot et al., (2012); Michot et al., 2010; Zeharia et al., 2008). Rhabdomyolysis is a condition characterized by the breakdown of damaged skeletal muscle and necrosis (Huerta‐Alardin et al., 2005; Zhang, 2012). Moreover, recent studies from ours and other research groups have observed a significant increase in centrally nucleated myofibers in different muscle‐specific lipin1‐deficient mouse models (Jama et al., 2019; Rashid et al., 2019; Schweitzer et al., 2019). Given that elevated centrally nucleated myofibers is a marker of myofiber death and regeneration, this demonstrates that loss of lipin1 plays a critical role in the muscle fiber degeneration.

There are three major types of muscle cell death: apoptosis, autophagic cell death, and necrosis (Sciorati et al., 2016). Previous studies from our and other groups have shown that lipin1 deficiency in mouse skeletal muscles blocks autophagy/mitophagy and therefore inhibits the induction of autophagic cell death (Alshudukhi et al., 2018; Zhang et al., 2014). Apoptosis is mediated by extrinsic and intrinsic signaling pathways. The intrinsic pathway is the major mechanism of apoptosis and has been detected in different muscular diseases, including muscular dystrophy (Bargiela et al., 2015; Sandri et al., 1998; Tidball et al., 1995), metabolic myopathy (Mirabella et al., 2000; Monici et al., 1998), and skeletal muscle denervation (Lee et al., 2017; Tews et al., 2005). In response to alterations in the ratio of antiapoptotic (such as Bcl‐2 and Bcl‐xl) and proapoptotic Bcl‐2 family proteins (such as Bax and Bak), or through the activation of initiator caspases (such as caspase‐9), intrinsic apoptosis can be initiated by the release of cytochrome c from the mitochondria to the cytoplasm, leading to the activation of caspase‐3 and nuclear DNA fragmentation (Elmore, 2007; Green & Llambi, 2015). Necroptosis is a genetically regulated form of necrotic cell death that has emerged as an important pathway in human disease (Linkermann et al., (2013); Ofengeim et al., 2015; Lin et al., 2013; Zhou & Yuan, 2014; Zhao et al., 2015; Bencze et al., 2017) and degeneration of myofibers (Morgan et al., (2018)). It is mediated by receptor‐interacting serine/threonine‐protein kinase 1 (RIPK1), RIPK3, and mixed‐lineage kinase domain‐like protein (MLKL; Vanden Berghe et al., 2016). RIPK3‐mediated MLKL phosphorylation is a key event in necroptosis execution that promotes MLKL oligomerization and subsequent assimilation into the plasma membrane, where it promotes membrane permeabilization and calcium influx (Morgan et al., 2018).

Loss of membrane integrity is the proximate cause of some muscular diseases such as Duchenne muscular dystrophy (DMD), inducing an enhanced myofiber death and muscle dysfunction in DMD (Gao and McNally, 2015). Previous studies from our and other laboratories have shown that a loss of lipin1 leads to increased central nucleation of myofibers, an upregulation of necroptotic gene expression, sarcoplasmic reticulum stress and loss of tetanic muscle force production (Alshudukhi et al., 2018; Jama et al., 2019; Rashid et al., 2019; Schweitzer et al., 2019). However, it remains unknown whether other cell death pathways such as apoptotic pathways are activated in lipin1‐deficient muscles and whether the cell death pathways are related with muscle membrane integrity.

In this study, we aimed to examine the involvement of apoptosis and/or necroptosis in the pathophysiology of muscle diseases characterized by the loss of lipin1. We examined changes in the expression of key apoptotic and necrotic makers in homozygous lipin1‐deficient muscles. We showed that the absence of lipin1**‐**induced apoptotic and necroptotic cell death in skeletal muscle. Moreover, we found that the loss of lipin1 is strongly associated with decreased muscle integrity and contributes to reduced muscle contractile force.

## 2 MATERIALS AND METHODS

### 2.1 Animals

Lipin1^Myf5cKO^ mice were generated by crossing Lipin1^fl/fl^ mice (Nadra et al., 2008) with Myf5‐Cre mice (Stock No: 007893; Jackson Laboratories) and have been reported in our recent study (Jama et al., 2019). Both Lipin1^fl/fl^ and Myf5‐Cre mice were in a C57/B6 background. Experiments were performed on 2‐ to 4‐month‐old mice, and only male mice were used in this study. General anesthesia used prior to tissue collection. These mice had free access to drinking water and regular chow. All animal experiments were performed in accordance with the relevant guidelines and regulations approved by the Animal Care and Use Committee of Wright State University.

### 2.2 Western blotting

Muscle tissues were isolated and snap frozen in liquid nitrogen for subsequent homogenization, or lysed in RIPA buffer (10 mm Tris‐HCL pH 7.4, 30 mm NaCl, 1 mm EDTA, 1% Nonidet P‐40) supplemented with proteinase inhibitors and phosphatase inhibitors before use. Protein concentration was determined for each sample and equal amounts of proteins were used, boiled for 5 min in 1× SDS sample buffer and separated by 7.5%–15% SDS‐PAGE. Thereafter, proteins were transferred to polyvinylidene difluoride membranes (Millipore) using a Mini Trans‐Blot Cell System (Bio‐Rad). The membrane was blocked with 1% casein buffer for 1 hr, and incubated with the primary antibodies overnight at 4°C. After probing with secondary antibodies for 1 hr at 25°C, protein bands were detected using Amersham Imager 600 (GE Healthcare Life Sciences). Primary antibodies used include lipin1 (#14906; Cell Signaling Technology), Bax (#2772), Bak (#12105), Bcl‐2 (#3498), Bcl‐xl (#2764), cleaved caspase 9 (ccp‐9, #9508), cleaved caspase 3 (ccp‐3, #9664), RIPK3 (#95702), RIPK1 (#3493T), and MLKL (#37705S). Goat anti‐mouse IgG‐HRP (#w402B; Promega, Madison, WI, USA) and goat anti‐rabbit IgG‐HRP (#w401B; Promega) secondary antibodies were used for detection. GAPDH (Ab181602; Abcam) antibody was used as a loading control. Western blots were quantified by densitometry using NIH Image J software and all values were normalized to a loading control.

### 2.3 Histological and immunohistochemical analysis

Lipin1^Myf5cKO^ and WT mice for each group were euthanized for histological or immunohistochemical analysis. Immediately following sacrifice, the gastrocnemius muscles were dissected, and frozen in 2‐methylbutane chilled to a slurry on liquid nitrogen. Sections of frozen tissue were prepared at 10 μm using a cryostat and stored at −20°C until stained. Hematoxylin and eosin staining were used for the analysis of mean myofiber cross‐sectional area and central nucleation, a hallmark of muscle degeneration/regeneration cycles accordingly to manufacturer's protocol (Vector Laboratories, H3502). The ratio of myofibers with centrally located nuclei to the total myofibers in the field was counted. For immunostainings, sections were blocked with 5% BSA‐PBS and incubated with the primary antibody. Negative controls were performed by omitting the primary antibody. Primary antibodies used in this study are laminin (Ab 11575, Abcam), Bax (#14796; Cell Signaling Technology), ccp3 (#9664), and RIPK3 (#95702). The primary antibody was incubated in humid chambers at 37°C for 1 hr, the slides were rinsed in PBS, and incubated in goat anti‐rabbit 488 secondary antibody (1:1,000; Invitrogen) for 1 hr. After a rinse in PBS, the slides were coverslipped with Vectashield mounting medium. Images were obtained using an inverted microscope (IX70; Olympus, Tokyo, Japan) equipped with a DFC7000T camera (Leica Microsystems, Wetzlar, Germany).

### 2.4 TUNEL staining

Frozen muscle sections were deparaffinized, rehydrated, fixed with 4% paraformaldehyde and permeabilized using 0.1% Triton X‐100 and 0.1% sodium citrate buffer. The permeabilized sections were incubated for 1 hr in TUNEL (terminal deoxynucleotidyl transferase dUTP nick end‐labeling) reaction mixture from an in situ cell death detection kit (Roche), washed, and incubated with Vectashield anti‐fade mounting medium with DAPI (Vector Laboratories, H‐1200).

### 2.5 Evans blue dye (EBD) assay

Mice were injected with EBD (10 mg/ml stock in sterile saline, 0.1 ml/10 g body weight) i.p. and euthanized 24 hr later. The skeletal muscles were dissected and snap‐frozen in isopentane cooled optimal cutting temperature (OCT) embedding media (Tissue‐Tek, Sakura‐Americas). Frozen OCT blocks were cryo‐sectioned at 10 µm thickness and stained with laminin antibody before being analyzed by fluorescence microscopy.

### 2.6 Creatine kinase assay

Serum creatine kinase (CK) activity was determined using an EnzyChrom™ Creatine Kinase Assay Kit (BioAssay Systems) according to the manufacturer's instructions. Briefly, 10 µl of mouse serum was added to 100 µl of reconstituted reagent into a 96‐well plate. To calibrate the data, calibrator, 10 µl, in 100 µl of water was used. Samples were incubated at room temperature. The rate of change in NADPH absorbance at 340 nm, which is proportionate to the CK activity, was measured after 20 and 40 min using Synergy H1 Hybrid Multi‐Mode Microplate Reader (Vermont, USA). CK activity was calculated by the following equation: [(*A *_340_)_4min_–(*A *_340_)_20mm_]/[(*A *_340_)_calibrator_–[(*A *_340_)_blank_] × 150.

### 2.7 Contractile force measurement

In vivo force measurements were performed on mice anesthetized with isoflurane using a SomnoSuite low‐flow anesthesia system (Kent Scientific), as reported (Wang et al., 2020). Body temperature was monitored with a SomnoSuite temperature probe and maintained at ~35°C using a heat lamp. Force was measured from the plantar flexor muscles (lateral and medial gastrocnemius, plantaris, and soleus muscles). During the procedure to expose the muscles and sciatic nerve, the common peroneal and tibial nerves were crushed to minimize the influence of adjacent muscles. The muscles were bathed in physiological saline during surgery to prevent them from drying out. Mice were then transferred to a custom 3D‐printed platform designed to eliminate movement during in vivo force recordings. The mouse limb was stabilized with vertical supports and by pinning the limb into recessed areas in the platform that was filled with Sylgard. The proximal end of the plantar flexor muscles (Achilles tendon) was attached to the lever of the force transducer (300D‐305C dual‐mode muscle lever, Aurora Scientific, Ontario, Canada) using 5–0 or 6–0 silk suture and a modified Miller's knot. To prevent drying, the exposed tissue was soaked in mineral oil during the contraction experiments. The full platform with the supported mouse was mounted to a micromanipulator (XR25/M, Thorlabs). To obtain optimal length, we determined the maximum twitch force while lengthening the muscle using the micromanipulator. Contractions were stimulated with platinum electrodes resting on the sciatic nerve. A/S900 Stimulator and S‐910 Stimulus Isolation Unit (Dagan Corp) were used to apply 1 ms suprathreshold voltage pulses. Muscle force was recorded and digitized using pClamp10 software (Molecular Devices).

### 2.8 Statistical analysis

Data are provided as the mean ± *SD* number (*n*) of independent experiments except for force measurement. Statistical significance was calculated using a two‐tailed Student's *t* test. Force‐frequency data were fit with the following Boltzmann equation,

$$y=\frac{F_{\min}‐F_{\max}}{1+e^{\left(\frac{x‐{\text{freq}}_{0.5}}{k}\right)}}+F_{\max},$$

where, *x* = frequency of stimulation, *y* = force, *F*_min_ = minimum force, *F*_max_ = maximum force, freq_0.5_ = frequency giving half‐max force, and *k* = slope factor. Data were analyzed with paired *t*‐tests using OriginPro 2019 or 2020 (OriginLab Corp., Northampton, MA) and reported as mean ± *SEM*. *p* < .05 was considered to be significant.

## 3 RESULTS

### 3.1 Fiber degeneration and regeneration in Lipin1^Myf5cKO^ muscle

**FIGURE 1:**
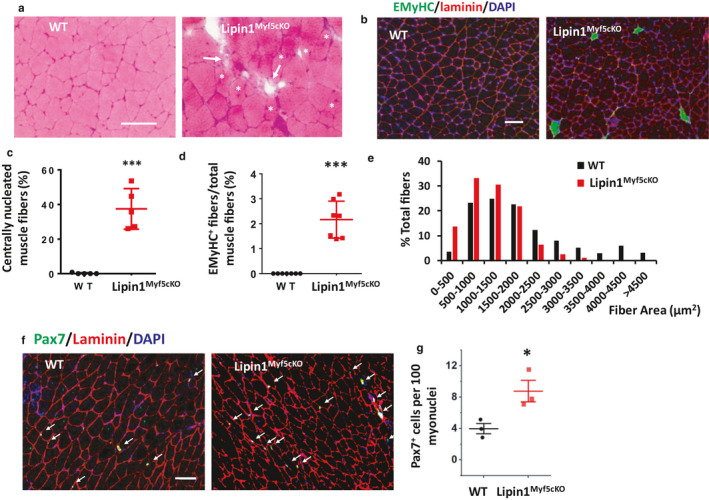
Enhanced muscle degeneration/regeneration in Lipin1^Myf5cKO^ mice. (a and b) H&E stained sections and eMyHC staining of gastrocnemius muscle from WT and Lipin1^Myf5cKO^ mice. Lipin1^Myf5cKO^ muscle showed myofiber necrosis labeled by arrows and centrally nucleated myofibers indicated by asterisks. (c and d) Percent of centrally nucleated fibers (*n* = 5 mice/group) and EMyHC‐stained fibers per total fiber number (*n* = 7 mice/group). (e) Histogram of muscle fiber size of gastrocnemius muscle. (f and g) Immunostaining and quantification analysis of Pax7‐positive satellite cells in gastrocnemius section of indicated mice. Pax7 immunostaining was shown in green, laminin in red, and nuclei were counterstained with DAPI (in blue). White arrows are indicating satellite cells (*n* = 3 mice/group). Scale bars = 100 µm. *p < .05; ***p < .005

We performed H&E histology (Figure 1a) and immunostaining for embryonic myosin heavy chain (EMyHC, green), laminin (red), and nuclei (blue) (Figure 1b) to examine degeneration and regeneration in Lipin1^Myf5cKO^ muscle. We observed degenerated myofibers in Lipin1^Myf5cKO^ muscle, which displayed characteristics such as pale muscle fibers with cytoplasmic disruptions (core, moth‐eaten, and targetoid fibers), regional hypercontraction, cellular infiltration, and myophagocytosis (white arrows, Figure 1a). We also observed central nucleated muscle fibers in Lipin1^Myf5cKO^ muscle (white stars, Figure 1a). Quantitative analysis of the H&E stained muscle showed that 37.46% of the Lipin1^Myf5cKO^ but no WT gastrocnemius fibers had central nuclei (Figure 1c, *p* = 1.0 × 10^−4^, *n* = 5 mice/group), indicating an increase in muscular degeneration and regeneration in Lipin1^Myf5cKO^ muscle. As shown in Figure 1b,d, 2.16% of the Lipin1^Myf5cKO^ fibers but none of the WT fibers expressed embryonic myosin (*p* = 5.45 × 10^−6^, *n* = 7 mice/group), which are consistent with increased fiber regeneration in Lipin1^Myf5cKO^ muscle. Finally, the fiber size distribution (Figure 1e) showed that there was a shift in smaller fibers in Lipin1^Myf5cKO^ mice, which are likely explained by the increase in regenerating muscle fibers. We also stained gastrocnemius sections from WT and from WT and Lipin1^Myf5cKO^ mice using a Pax7 antibody and found that lipin1 deficiency leads to an increased number of satellite cells (Figure 1f,g). This is consistent with the elevated muscle degeneration/regeneration cycle we observed in lipin1‐deficient muscle. However, our previous publications identified that lipin1 deficiency leads to impaired muscle regeneration (Jiang et al., 2015) and decreased MyoD and MEF2c expression (Jama et al., 2019). Therefore, it is possible that upregulation of Pax7 could be due to both elevated muscle degeneration/regeneration and compensation for impaired myoblast differentiation process.

### 3.2 Apoptosis in Lipin1^Myf5cKO^ muscle

**FIGURE 2:**
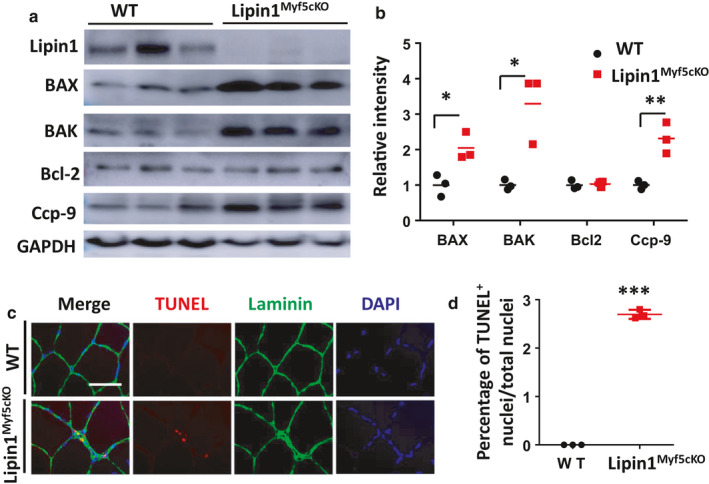
Western blot analysis of apoptosis‐related proteins and TUNEL assay in gastrocnemius from WT and Lipin1^Myf5cKO^ mice. (a) Western blot assays of proapoptotic (Bax and Bak), antiapoptotic markers (Bcl‐2), and cleaved caspase‐9 (Ccp‐9) in gastrocnemius of WT and Lipin1^Myf5cKO^ mice. (b) Densitometry analysis of Western blot images is presented as mean ± *SD* from three independent experiments. (c) Immunofluorescence assessment of TUNEL staining in gastrocnemius of WT and Lipin1^Myf5cKO^ mice. Scale bar = 50 μm. The blue‐strained cells are DAPI; the red‐strained cells are TUNEL‐positive cells. (D) Quantification of TUNEL‐positive cells (*n* = 3 mice/group). *p < .05; **p < .01, ***p < .005, Lipin1^Myf5cKO^ versus WT measured by *t*‐test

To determine whether the muscle degeneration in Lipin1^Myf5cKO^ mice was due to the induction of apoptotic or necrotic cell death, we first evaluated the changes in apoptosis‐related protein expression by Western blotting and examined the TUNEL‐labeled cells under a fluorescence microscope. As shown in Figure 2a,b, the proapoptotic protein Bax and Bak showed marked increases (205% and 330% with *p* = .02 respectively, *n* = 3 mice/group) in gastrocnemius of Lipin1^Myf5cKO^ mice compared to WT mice. The detection of cleaved caspase‐9 (ccp‐9) by western blotting is evidence of the activation of caspase‐9. A significant increase (231%, *p* = .007) of ccp‐9 in Lipin1^Myf5cKO^ muscle was also observed compared with WT controls. The expression of the antiapoptotic protein Bcl‐2 was no difference in Lipin1^Myf5cKO^ muscles compared with WT controls.

We also detected the apoptotic DNA fragmentation using the TUNEL method. Double labeling TUNEL/laminin‐detected nuclei positive for DNA fragmentation in gastrocnemius of lipin1‐deficient muscles, but detected very few in WT samples (Figure 2c,d). Quantitative measurement of apoptotic nuclei revealed that the frequency of TUNEL‐positive myonuclei increased to 2.7% in Lipin1^Myf5cKO^ mice (*p* = 9.48 × 10^−7^, *n* = 3 mice/group). Moreover, we noticed that apoptosis was present both in interstitial cells and myofibers in lipin1‐deficient mice.

### 3.3 Necroptosis in Lipin1^Myf5cKO^ muscle

**FIGURE 3:**
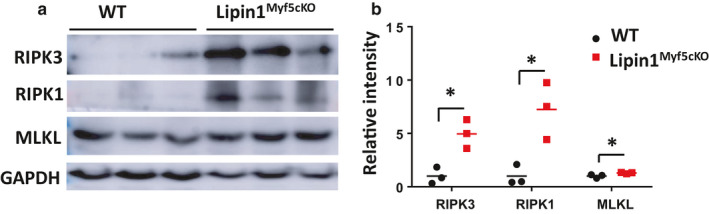
Western blot analysis of necroptosis markers in gastrocnemius from WT and Lipin1^Myf5cKO^ mice. (a) Representative immunoblots and (B) quantitative analysis of RIPK1, RIPK3, and MLKL expression (*n* = 3 mice/group). *p < .05

We investigated the presence of necroptosis in Lipin1^Myf5cKO^ muscles. The upregulated expression of proteins belonging to the necroptosis machinery (i.e., RIPK1, MLKL, and RIPK3) is a strong indication of necroptosis. In the gastrocnemius of Lipin1^Myf5cKO^, the expression of necroptotic proteins, MLKL, RIPK1, and RIPK3, were significantly increased by 130% (*p* = .047), 724% (*p* = .018), and 496% (*p* = .011) respective as compared to values from WT mice (Figure 3a,b, *n* = 3 mice/group).

### 3.4 Plasma membrane breakdown in Lipin1^Myf5cKO^ muscle

**FIGURE 4:**
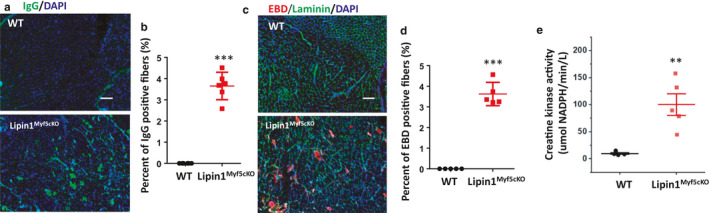
IgG staining and Evans blue dye staining in gastrocnemius sections indicate myofiber damage in Lipin1^Myf5cKO^ mice. (a) gastrocnemius muscle sections from WT and Lipin1^Myf5cKO^ mice were immunostained with goat anti‐mouse IgG to detect permeable/damaged fibers. (b) Quantification (%) of IgG‐positive fibers (*n* = 6 mice/group). (c) Representative images of EBD‐positive staining and (d) the percentage of EBD‐positive necrotic fibers in Lipin1^Myf5cKO^ mice (*n* = 5 mice/group). Scale bar = 200 µm. (e) serum creatine kinase levels in indicated mice. **p < .01; ***p < .005

Loss of plasma membrane integrity is a hallmark of necrotic cell death. To identify damaged skeletal muscle fibers, we stained them with the membrane‐impermeable marker IgG. As shown in Figure 4a,b, gastrocnemius muscles from Lipin1^Myf5cKO^ mice demonstrated that 3.65% of total muscle fibers was IgG‐positive compared to zero in WT muscle (*p* = 7.7 × 10^−8^, *n* = 6 mice/group), suggesting the loss of membrane integrity. To confirm that these muscle fibers were indeed damaged, we injected EBD into the Lipin1^Myf5cKO^ and WT mice. As shown in Figure 4c,d, Individual EBD‐positive muscle fibers were scattered throughout the muscle sections of Lipin1^Myf5cKO^ mice, but not in WT mice. Quantification analysis showed that 3.62% of EBD‐positive fibers was detected in gastrocnemius of Lipin1^Myf5cKO^ mice, but none in WT mice (*p* = 5.42 × 10^−7^, *n* = 5 mice/group).

Serum creatine kinase levels are an important clinical indicator of muscle damage in various medical conditions including muscular dystrophy and myocardial infarction. (Duma & Siegel, 1965; Gaines et al., 1982; Hathout et al., 2016; Somer et al., 1975). We therefore examined the serum creatine kinase activity in Lipin1^Myf5cKO^ and WT mice by a colorimetric assay (Figure 4e). We found that creatine kinase activity was increased to 100.08 µmol NADPH/min/L in Lipin1^Myf5cKO^ mice compared to 9.69 µmol NADPH/min/L in WT mice (*p* = .002, *n* = 5 mice/group), indicating the presence of muscle damage in Lipin1^Myf5cKO^ mice.

### 3.5 Myofiber death is associated with plasma membrane disruption

**FIGURE 5:**
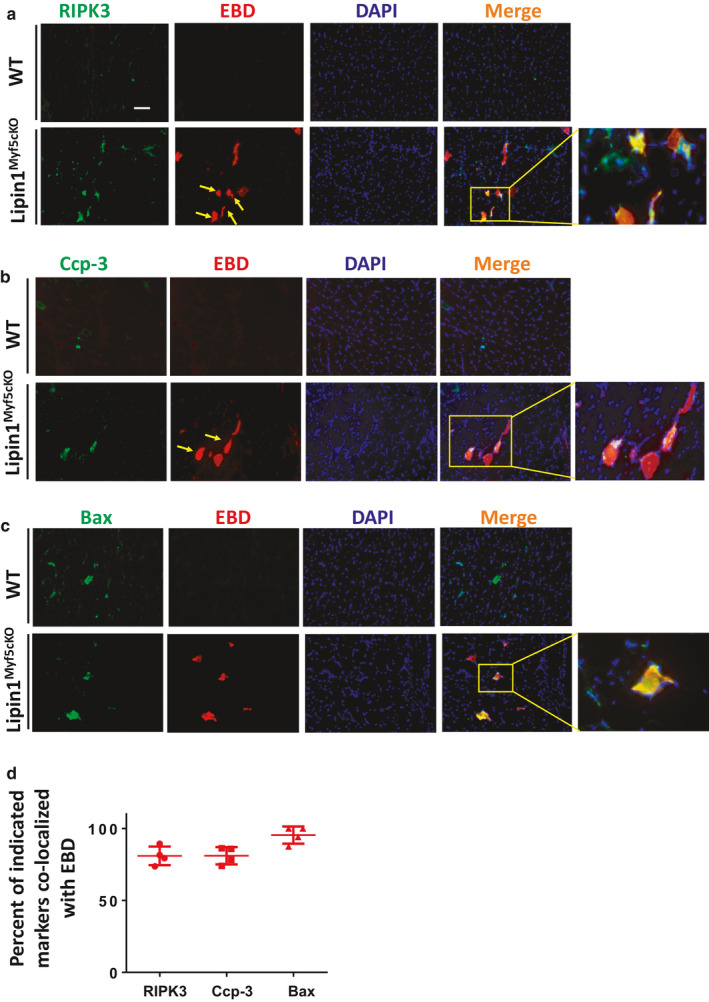
Colocalization of Evans blue dye staining with RIPK3, cleaved caspase 3, and Bax in gastrocnemius sections in Lipin1^Myf5cKO^ mice. Gastrocnemius muscles from WT and Lipin1^Myf5cKO^ mice were collected from WT 1‐day post‐injection of EBD, and immunostained with either primary antibodies against (a) RIPK3, (b) cleaved caspase 3 (Ccp‐3) or (c) Bax. (d) Colocalization of EBD with RIPK3, cleaved caspase 3 or Bax in WT, and Lipin1^Myf5cKO^ muscle were examined and quantified (*n* = 4 mice/group). Scale bar = 100 µm

To determine whether loss of membrane integrity could be induced in necroptotic or apoptotic myofibers, we examined the co‐localization of RIPK3 and cleaved caspase‐3 (ccp‐3) with damaged EBD‐positive myofibers by immunoblotting (Figure 5). We found an intense co‐localization between EBD‐positive staining with RIPK3 (Figure 5a), cleaved ccp‐3 (Figure 5b) and Bax (Figure 5c) in muscles from Lipin1^Myf5cKO^ mice. Approximately, 80.94% of RIPK3‐positive muscle fibers and 81% of ccp‐3‐positive myofibers were‐EBD positive (Figure 5d, *n* = 4 mice/group). Interestingly, among the Bax‐positive myofibers, 95.4% myofibers were EBD‐positive suggesting that membrane damage may lead to the initiation of apoptosis. These data show a strong link between loss of membrane integrity and necrotic/apoptotic cell death and support the implication that membrane disruption may lead to necroptotic/apoptotic myofibers.

### 3.6 Decreased contractile force in Lipin1^Myf5cKO^ muscle

**FIGURE 6:**
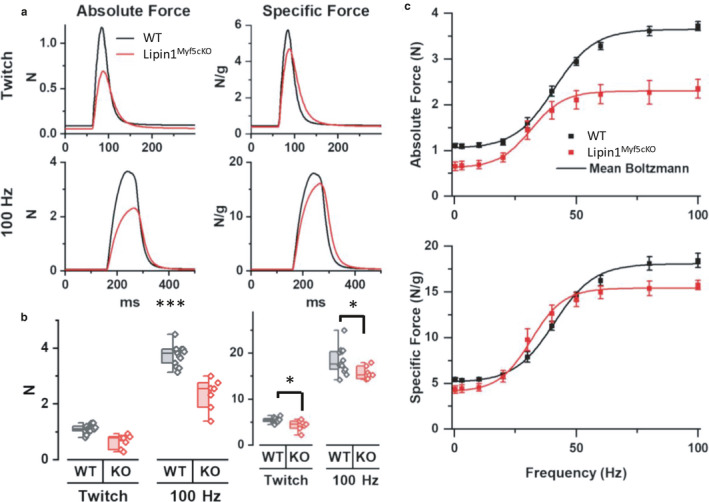
Lipin1 deficiency leads to reduced contractile force in gastrocnemius of Lipin1^Myf5cKO^ mice. (a) Average absolute (left column) and specific (right column) force traces from WT (black) and Lipin1^Myf5cKO^ (red) muscle with twitches shown in the top panels and maximum fused tetanic contractions (100 Hz stimulation) in the bottom panels. (b) Half box plots of the peak force during twitch and 100 Hz maximum contractions of WT (black) and Lipin1^Myf5cKO^ (KO, red), measured as absolute force (left panel) and specific force (right panel). The box on the left half indicates the 25%–75% interquartile range, the whiskers indicate 1.5 times the interquartile range, and the bar indicates the median. Each data point is shown in the right half. *p < .05; ***p < .005. (c) Average force‐frequency data with the Boltzmann fit (solid line) for WT (black) and Lipin1^Myf5cKO^ (red) muscle. Data are shown for the absolute force (top panel) and specific force (bottom panel). Values shown as ± *SEM*, *n* = 13 WT and 7 Lipin1^Myf5cKO^ mice

We assessed muscle function of WT (*n* = 13) and Lipin1^Myf5cKO^ (*n* = 7) mice by measuring in vivo isometric force of the plantar flexor (medial and lateral gastrocnemius, plantaris, and soleus) muscles in response to sciatic nerve stimulation. The average WT and Lipin1^Myf5cKO^ muscle force in response to a single action potential (twitch) and during a maximal fused tetanic contraction (100 Hz stimulation) are shown in the left panel of Figure 6a. The maximal tetanic force was measured in response to 10 or 15 stimulus pulses at 100 Hz. The traces in Figure 6 were averaged from the 100 Hz train with 10 stimulus pulses (*n* = 5 WT and 6 Lipin1^Myf5cKO^). To account for the significantly lower mass of the Lipin1^Myf5cKO^ plantar flexor muscles (0.148 ± 0.010 g) compared to WT (0.205 ± 0.009 g, *p* = 6.8 × 10^−4^), the absolute forces were normalized by muscle weight to obtain the specific force (Figure 6a, right column). The average peak force during twitch and 100 Hz tetanic contractions in WT (*n* = 13) and Lipin1^Myf5cKO^ (*n* = 7) muscle are shown in Figure 6b. The absolute twitch force and 100 Hz max force were significantly lower in Lipin1^Myf5cKO^ compared to WT muscle (Figure 6b, twitch *p* = 7.4 × 10^−5^ and 100 Hz *p* = 2.1 × 10^−4^ with Welch correction for unequal variance). Much of this difference in force was due to the reduced muscle mass of the Lipin1^Myf5cKO^ muscle. However, the significant reduction in Lipin1^Myf5cKO^‐specific force compared to WT of the twitch (*p* = .041 with Welch correction for unequal variance) and 100 Hz tetanic (*p* = .033) contractions suggests a disruption in excitation‐contraction coupling in the Lipin1^Myf5cKO^ muscle.

We also measured force in response to a full range of physiologically relevant stimulation frequencies (0.3–100 Hz); at each frequency, 10 or 15 stimulus pulses were stimulated. The average absolute peak force in Lipin1^Myf5cKO^ muscle was reduced compared to WT throughout the frequency range tested (Figure 6c, top panel) and the average peak‐specific force in Lipin1^Myf5cKO^ muscle was reduced at low and high frequencies of stimulation (Figure 6c, lower panel). In addition, the frequency that produced of half‐maximal force was significantly lower in Lipin1^Myf5cKO^ (31 ± 2 Hz) compared to WT (41 ± 2 Hz) muscle (*p* = .005). The leftward shift in the force–frequency relationship of Lipin1^Myf5cKO^ muscle suggests a fast‐to‐slow fiber type shift.

## 4 DISCUSSION

Our data revealed that the absence of lipin1 induces not only caspase‐dependent intrinsic apoptosis, but also RIPK3‐dependent necroptosis in skeletal muscle. Membrane damage is associated with the activation of apoptotic and necroptotic pathways. Understanding the signaling events that regulate the initiation and development of skeletal muscle fiber loss will allow for the development of novel treatment strategies for these muscle disorders.

Furthermore, the level of muscle degeneration and regeneration in Lipin1^Myf5cKO^ mice was substantial, with Lipin1^Myf5cKO^ muscle having 37% of its fibers with central nuclei and 2% expressing embryonic myosin heavy chain suggesting that Lipin1^Myf5cKO^ muscles undergo frequent degeneration–regeneration cycles. This is consistent with observations in previous studies using either human skeletal actin (HSA; Rashid et al., 2019), or muscle creatine kinase (MCK; Schweitzer et al., 2019) promoters generated skeletal muscle‐specific lipin1‐deficient mice. This pathology likely explains the decreased mass of Lipin1^Myf5cKO^ muscle found here and in our previous report (Jama et al., 2019).

Our data provide the first evidence that apoptotic and necroptotic cell death co‐exist in Lipin1^Myf5cKO^ muscle. There are three major types of cell death: apoptosis, autophagic cell death, and necrosis. Zhang's and our recent studies reported that lipin1 deficiency in mice is associated with a blockade in autophagic flux, a deregulated mitophagy and a marked accumulation of abnormal mitochondria and autophagic vacuoles in skeletal muscle (Alshudukhi et al., 2018; Zhang et al., 2014). In this study, we observed the elevations of TUNEL‐positive nuclei and apoptotic DNA fragmentation in Lipin1^Myf5cKO^ muscles. We further demonstrated the Bax and Bak protein signaling and activation of caspase‐9 pathway are involved in the underlying mechanism of muscle loss in Lipin1^Myf5cKO^ mice. Our data indicated that lipin1 deficiency induced a marked increase in proapoptotic Bax, Bak, and cleaved caspase 9 protein content. We also found upregulated expression of RIPK1, MLKL, and especially RIPK3 in skeletal muscles of Lipin1^Myf5cKO^ mice, indicating necroptosis activation induced by lipin1 deficiency, which is in line with enhanced mRNA expression of these three genes reported by Schweitzer et al. (2019). On the other hand, apoptosis and necrosis can coexist in the degenerating/regenerating muscles, including those of patients with neuromuscular disorders, such as inflammatory myopathies, dystrophies, metabolic, and mitochondrial myopathies and drug‐induced myopathies (Sciorati et al., 2016).

Most importantly, our data further showed that loss of membrane integrity is strongly associated with necroptosis and apoptosis. It has been proposed that the initial event in muscle cell necrosis in a variety of pathological conditions including DMD is the focal breakdown of the plasmalemma (Carpenter & Karpati, 1979; Mokri & Engel, 1998; Schmalbruch, 1975; Weller et al., 1990). Membrane rupture allows the influx of calcium and leads to necrotic muscle fiber death. Our data showed approximately 81% RIPK3 myofibers were EBD‐positive, suggesting the disruption of membrane integrity occurs relatively early and may lead to necroptosis. Although our results suggest the association between membrane damage and myofiber death, future investigation is necessary to identify causality. In the future, we will ablate these proteins in Lipin1^Myf5cKO^ mice to examine whether muscle membrane integrity can be improved in lipin1‐deficient muscles through the inhibition of apoptotic or necroptotic pathways.

Our data also suggest that loss of lipin1 is related to compromised muscle membrane integrity. Lipin1 is a Mg^2+^‐dependent phosphatidate phosphatase (PAP1) enzyme catalyzing the dephosphorylation of phosphatidic acid, yielding diacylglycerol which are lipid precursors used for the synthesis of the major membrane phospholipids phosphatidylethanolamine and phosphatidylcholine. Phosphatidylethanolamine and phosphatidylcholine are not only important for membrane structure, fluidity, and stability (Dawaliby et al., 2016; Li et al., 2006), but also affect membrane protein trafficking (Schuler et al., 2016; Testerink et al., 2009). Lipin1 deficiency may affect membrane integrity through altering membrane phospholipid contents and affecting membrane protein expression. Moreover, our previous study identified a formerly unknown role of lipin1 in promoting myocyte enhancer factor 2c (MEF2c) transcriptional activity through DAG‐mediated signaling (Jama et al., 2019). MEF2c has been shown to regulate a wide range of sarcolemmal membrane structural genes and directly regulates myofiber integrity by targeting genes that encode a network of structural proteins (Blais et al., 2005; Potthoff et al., 2007; Sartorelli et al., 1993). This study demonstrates that lipin1 may play an important role in maintaining myofiber stability and integrity.

Moreover, it is generally accepted that loss of membrane integrity occurs late in apoptosis (Nelson et al., 2011), (Rogers et al., 2017). Interestingly, we observed proapoptotic marker Bax‐positive muscle fibers highly co‐localized with EBD‐positive muscle fibers, suggesting that loss of membrane integrity may contribute to the early stages of apoptosis. This is consistent with a previous study which demonstrated that increased membrane permeability may induce calcium influx and eventually trigger apoptosis (Matsuda et al., 1995). We propose that in Lipin1^Myf5cKO^ muscles, high levels of Ca2+ entry due to cell membrane damage triggers cell death via necrosis or apoptosis, depending the severity of the damage. A previous study has identified that exercise training was effective in diminishing apoptosis in aging heart (Kwak, 2013). It is possible that appropriate exercise training could be beneficial to patients with lipin1 deficiency by inhibiting apoptosis and necroptosis.

As a consequence of muscle degeneration, a marked reduction in total muscle force was observed, which is consistent with previous studies using global lipin1‐deficient fatty liver dystrophy fld mice (Jiang et al., 2015), and HAS^Cre/+^/Lpin1^fEx34/fEx3‐411^ mice (Rashid et al., 2019). When isometric forces were corrected for the muscle mass (the specific force, N/g), there was still a significant difference in force between Lipin1^Myf5cKO^ and control muscle, indicating a defect in the Lipin1^Myf5cKO^ muscle that is in addition to the muscle mass loss. Indeed, previous studies have reported that sarcolemma disruption has been implicated in the reduction in muscle force after muscle injury (Hamer et al., 2002; McNeil & Khakee, 1992; Sam et al., 2000; Tidball, 2002). Lovering & De Deyne identified a complementary correlation between maximal tetanic tension and EBD‐positive muscle fibers suggesting that membrane integrity is critical for the maintenance of muscle contractile force (Lovering & De Deyne, 2004). Enhanced apoptosis (Schwartz & Ruff, 2002) and necroptosis (Morgan et al., 2018) could change contractile properties and leads to reduced contractile force production.

Lastly, we examine the limitations of this study and identify areas for future research. Despite revealing that lipin1 plays an important role in protecting against myofiber degeneration and maintain membrane integrity, more investigations are necessary to elucidate how lipin1 deficiency leads to membrane damage. Inadequate membrane repair has been implicated to participate in the pathogenesis of muscular dystrophies and cardiomyopathies (Han et al., 2007; Lammerding & Lee, 2007; Vila et al., 2017). Another future direction would be to examine whether restoration of lipin1 in lipin1‐deficient muscle could improve membrane integrity. Furthermore, we have determined lipin1 deficiency induces myocyte apoptosis and necrosis. This enables further investigation into whether myocyte apoptosis and necrosis are two separate processes or a continuum of events and the conditions the cell chooses between different types of cellular deaths or survival.

## 5 CONCLUSIONS

In summary, we found that loss of plasma membrane integrity due to lipin1 deficiency in skeletal muscle fibers may play a primary role in the death of muscle fibers and the course of related muscle disorders. We demonstrated that lipin1 plays an important role in suppressing apoptotic and necrotic muscle cell death and preventing the muscle damage. We also demonstrated that muscle loss from atrophy and degeneration induced by lipin1 deficiency results in impaired force production. We propose that lipin1‐mediated muscle degeneration and higher muscle membrane vulnerability are one of the potential mechanisms that lead to muscle weakness. Our data provide a novel point for understanding of the mechanisms that regulate the muscle cell death, which could be utilized for the effective treatment of related muscle diseases.

## CONFLICT OF INTEREST

The authors declare that they have no conflict of interests.

## AUTHOR'S CONTRIBUTIONS

H. R. and A.A.V. designed the experiments; S. R. S., R. R., A. J., A.A., E. M. H., and R.R performed the research; N.E.T. and D. R. M. generated muscle force data; S. R. S., A. J., D. H., D. R. M., A. A. V., and H. R. analyzed the data; H. R. and A.A.V. wrote the manuscript; and all authors read and approved the manuscript.
